# JAK Inhibitors and Risk of Cancer in IBD Patients

**DOI:** 10.3390/cancers17111795

**Published:** 2025-05-28

**Authors:** Francesca Bernardi, Ilaria Faggiani, Tommaso Lorenzo Parigi, Alessandra Zilli, Mariangela Allocca, Federica Furfaro, Laurent Peyrin-Biroulet, Silvio Danese, Ferdinando D’Amico

**Affiliations:** 1 Gastroenterology and Endoscopy IRCCS, Ospedale San Raffaele, Via Olgettina 60, 20132 Milano, Italy; bernardi.francesca@hsr.it (F.B.); faggiani.ilaria@hsr.it (I.F.); parigi.tommaso@hsr.it (T.L.P.); zilli.alessandra@hsr.it (A.Z.); allocca.mariangela@hsr.it (M.A.); furfaro.federica@hsr.it (F.F.); danese.silvio@hsr.it (S.D.); 2 Gastroenterology and Endoscopy, Vita Salute San Raffaele University, Via Olgettina 60, 20132 Milano, Italy; 3 Department of Gastroenterology, INFINY Institute, INSERM NGERE, CHRU Nancy, F-54500 Vandœuvre-lès-Nancy, France; peyrinbiroulet@gmail.com

**Keywords:** inflammatory bowel disease, ulcerative colitis, Crohn’s disease, JAK inhibitor, cancer, tofacitinib, filgotinib, upadacitinib

## Abstract

Janus kinase inhibitors, including tofacitinib, filgotinib, and upadacitinib, have emerged as effective therapeutic options for the management of inflammatory bowel diseases (IBDs). By targeting the JAK-STAT signaling pathway, these agents modulate immune responses and reduce inflammation. However, concerns regarding the potential risk of malignancy associated with their use have gained significant attention. The JAK-STAT pathway is not only critical for inflammatory signaling but also plays a pivotal role in cellular growth, differentiation, and tumor surveillance. Observational studies and clinical trial data in rheumatoid arthritis have reported malignancies, including non-melanoma skin cancer and solid tumors, in patients receiving JAK inhibitors, with evidence suggesting variable risks depending on the selectivity of the agent. Current evidence does not suggest an increased risk of oncogenesis in patients with IBDs. Balancing therapeutic efficacy with long-term safety requires ongoing vigilance; patient stratification based on risk factors; and tailored monitoring strategies to mitigate potential adverse effects, including malignancies, during JAK inhibitor therapy. Long-term follow-up data of up to 10 years offer reassuring evidence that JAK inhibitor therapy in IBD patients does not confer an increased risk of malignancies, supporting their continued use within appropriate clinical settings.

## Simple Summary

Inflammatory bowel diseases (IBDs), such as Crohn’s disease and ulcerative colitis, are chronic conditions that cause inflammation in the digestive tract. New treatments called Janus kinase (JAK) inhibitors—like tofacitinib, filgotinib, and upadacitinib—have shown strong effectiveness in controlling symptoms by targeting key pathways in the immune system. However, there have been concerns about whether these drugs might increase the risk of cancer. This review explores the available data on the safety of JAK inhibitors, especially regarding cancer risk, and finds no clear evidence of increased malignancy in IBD patients. By reviewing clinical studies and long-term follow-up data, this work aims to help guide the safe and informed use of these medications. The findings offer reassurance for both clinicians and patients, supporting the continued use of JAK inhibitors in IBD treatment when appropriate safety measures and patient monitoring are in place.

## 1. Introduction

The advent of Janus kinase (JAK) inhibitors has revolutionized the therapeutic landscape for a variety of chronic inflammatory and autoimmune conditions [1]. These small-molecule drugs, originally developed for rheumatoid arthritis (RA) [2], have gained traction in the management of inflammatory bowel diseases (IBDs), including ulcerative colitis (UC) and Crohn’s disease (CD) [3,4].

The mechanism of action of JAK inhibitors lies in their ability to modulate the JAK-STAT (signal transducer and activator of transcription) signaling pathway [5]. This pathway is integral to the intracellular transduction of signals from cytokine receptors, which regulate immune cell activation, proliferation, and inflammatory responses [6]. By selectively inhibiting one or more of the JAK family enzymes (JAK1, JAK2, JAK3, and TYK2), JAK inhibitors block the downstream effects of pro-inflammatory cytokines such as interleukin-6 (IL-6), interferon-gamma (IFN-γ) and tumor necrosis factor-alpha (TNF-α) [7]. This targeted suppression of inflammatory signaling helps restore immune homeostasis and reduce the symptoms of chronic inflammation in IBD patients [7].

Within the IBD therapeutic landscape, tofacitinib, filgotinib, and upadacitinib stand out as key JAK inhibitors, each with distinct characteristics regarding their indications and mechanisms of action.

Tofacitinib was the first JAK inhibitor approved for use in moderate-to-severe UC [3,8]. Unlike its counterparts, tofacitinib is a pan-JAK inhibitor, targeting JAK1, JAK2, and JAK3 [9]. This broad inhibition allows it to modulate a wide array of pro-inflammatory cytokines, such as IL-6, IFN-γ, and interleukin-2 (IL-2), which are implicated in the pathogenesis of UC [10]. It has been approved for the treatment of RA in patients with an inadequate response to methotrexate and indicated as a treatment for psoriatic arthritis (PsA) and ankylosing spondylitis (AS), where it has demonstrated efficacy in reducing disease activity and improving patient-reported outcomes [11,12,13]. Furthermore, it is being explored in other autoimmune conditions such as systemic lupus erythematosus (SLE), though its use in these indications remains off-label or investigational [14].

In contrast, filgotinib is a selective JAK1 inhibitor, designed to specifically target pathways more directly involved in inflammatory signaling while sparing JAK2- and JAK3-mediated pathways, which are crucial for hematopoiesis and immune surveillance [15]. This selectivity is thought to reduce the risk of side effects associated with pan-JAK inhibition [15]. Filgotinib has shown efficacy in clinical trials for UC, while it is not approved for CD [16]. Its selective mechanism of action may offer a more favorable safety profile compared to tofacitinib, particularly in terms of infections and hematologic effects [17]. It is approved for RA, particularly in patients who have failed conventional disease-modifying antirheumatic drugs (DMARDs) [18,19]. Studies are ongoing to evaluate its potential in other conditions, such as PsA and AS, but its use in these areas is not yet widely established [20,21].

Upadacitinib, another selective JAK1 inhibitor, has emerged as a next-generation option for the treatment of IBDs, including both UC and CD [22,23]. Its high selectivity for JAK1 enhances its ability to suppress key inflammatory cytokines like IL-6 and IFN-γ without significantly affecting pathways mediated by JAK2 or JAK3 [15]. Upadacitinib has been shown to induce and maintain clinical remission in UC and CD [22,23]. Compared to tofacitinib, upadacitinib’s selective inhibition may result in fewer systemic side effects, although long-term safety data are still being collected. It is approved for RA, where it has shown superior efficacy compared to adalimumab in head-to-head trials [24,25]. Upadacitinib has also been indicated for the treatment of PsA and AS, providing an alternative to biologics for patients with these conditions [24,26,27]. Additionally, it has been approved for atopic dermatitis (AD), where its potent anti-inflammatory effects help manage severe disease [24]. This indication sets upadacitinib apart from tofacitinib and filgotinib, as it highlights its broader applicability in dermatologic conditions [28]. Furthermore, ongoing trials are exploring its efficacy in conditions such as SLE and giant cell arteritis, expanding its potential therapeutic scope [29,30].

In 2022, an RCT published in *The New England Journal of Medicine* found that the incidence of cancers was higher with tofacitinib compared to tumor necrosis factor (TNF) inhibitors in patients with RA older than 50 years of age and had at least one additional cardiovascular risk factor [31]. Over a median follow-up period of 4.0 years, the overall incidence of cancers (excluding non-melanoma skin cancer (NMSC)) was greater in patients receiving combined doses of tofacitinib (4.2%; 122 cases) compared to those treated with a TNF inhibitor (2.9%; 42 cases) [31]. Specifically, the study reported a higher occurrence of lung cancer and lymphoma among tofacitinib users [31]. It is nevertheless important to emphasize how, in subgroup analyses, the disparity in the risk of cancer between tofacitinib and TNF inhibitors was more evident among patients aged 65 years or older compared to those in younger age groups [31].

This led regulatory agencies like the FDA and EMA to issue warnings about its use in high-risk populations. The FDA highlighted an increased cancer risk with Xeljanz/Xeljanz XR (tofacitinib), particularly lymphomas and lung cancers, especially in current or past smokers. Patients with a history of malignancies or those developing cancer during treatment may also be at heightened risk. The FDA advises reserving tofacitinib for patients unresponsive or intolerant to TNF blockers, emphasizing careful risk–benefit assessment for individual patients [32].

**Figure 1:**
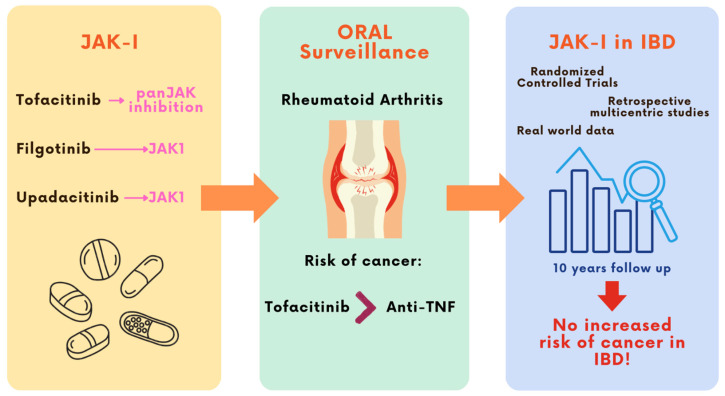
JAK inhibitors have emerged as effective therapeutic agents for the treatment of IBDs. However, following the publication of the ORAL Surveillance trial in 2022—reporting an increased risk of malignancies with tofacitinib in older RA patients with cardiovascular risk factors—concerns were raised regarding their oncologic safety. This led to regulatory warnings in high-risk populations. Nonetheless, data from IBD cohorts with up to 10 years of follow-up have not confirmed this increased cancer risk, providing reassuring evidence specific to the IBD population.

Although these findings triggered concerns about the safety of JAK-I in relation to malignancy risk, all subsequent evidence from studies in IBD patients over the years has not corroborated the signal reported by the ORAL Surveillance trial [Figure 1].

This review aims to provide a comprehensive and up-to-date synthesis of the available evidence regarding the potential malignancy risks associated with the use of JAK inhibitors in IBD treatment, with a focus on long-term follow-up data and molecule-specific safety profiles. By doing so, it seeks to support informed clinical decision-making and contribute a nuanced understanding of the evolving safety landscape of JAK inhibitors in IBD treatment.

## 2. JAK-I and Risk of Cancer

The same mechanism that underpins the therapeutic efficacy of JAK inhibitors may also influence specific signaling pathways involved in the regulation of oncogenesis [32]. The JAK-STAT pathway is not only crucial for inflammatory signaling but also plays a pivotal role in cellular growth, survival, and differentiation [33]. The dysregulation of this pathway has been implicated in carcinogenesis, with evidence suggesting that prolonged inhibition of JAK signaling could impair tumor surveillance mechanisms, such as natural killer (NK) cell activity, or disrupt normal apoptosis [34]. Prolonged or extensive inhibition of this pathway may impair NK cell function, which is essential for the detection and elimination of aberrant or malignant cells [34]. NK cells rely on JAK-STAT-mediated responses to cytokines such as interleukin-15 (IL-15) for their activation, and the disruption of this signaling could attenuate their tumor surveillance capabilities [34]. Additionally, the suppression of apoptosis through dysregulated signaling may allow for the survival and expansion of potentially oncogenic cells [33].

The degree of cancer risk appears to vary with the selectivity of the JAK-I; pan-JAK inhibitors, such as tofacitinib, which target multiple JAK isoforms (JAK1, JAK2, JAK3, and TYK2), may potentially modulate a wider spectrum of molecular signaling pathways compared to more selective inhibitors like filgotinib and upadacitinib, which predominantly target JAK1 [17,34]. This variation in risk underscores the importance of the selective modulation of JAK signaling to balance therapeutic efficacy with the potential for adverse effects. Overall, the immunosuppressive properties of JAK-I, particularly their effect on NK cell-mediated immune surveillance and apoptosis regulation, may create a permissive environment for carcinogenesis, necessitating careful risk–benefit assessment and monitoring in clinical practice [32].

## 3. Tofacitinib

Tofacitinib, approved for the treatment of UC, has been extensively studied in the OCTAVE Induction 1 [35] and 2 [35], OCTAVE Sustain [36] and OCTAVE Open trials, three phase III, randomized, double-blind, placebo-controlled trials. Its efficacy in inducing and maintaining remission and its safety profile in UC patients is well documented.

In the induction trials, two cases of NMSC were reported at the 8-week treatment mark [35]. One case involved a patient in the 10 mg tofacitinib group of the OCTAVE Induction 1 trial, who had a prior history of NMSC and was diagnosed with squamous cell carcinoma of the skin [35]. The second case involved a patient in the 10 mg tofacitinib group of the OCTAVE Induction 2 trial, who was diagnosed with basal cell carcinoma of the skin [35]. No cases of malignancies other than NMSC were observed in the induction trials [35].

In the OCTAVE Sustain trial, four cases of NMSC were reported at the 52-week mark [36]. Among these, three patients in the 10 mg tofacitinib group, all with a prior history of NMSC, were diagnosed with squamous cell carcinoma (*n* = 2) or basal cell carcinoma (*n* = 1) [36]. Additionally, one patient in the placebo group was diagnosed with basal cell carcinoma [36]. All patients who developed NMSC had a history of prior exposure to thiopurines [36]. However, one patient developed invasive ductal breast carcinoma, but this patient received a placebo during both the induction and maintenance phases of the trial [36].

A higher incidence of NMSC was observed in patients receiving tofacitinib compared to those on placebo across the OCTAVE trials [37]. This finding aligns with the previously reported association between tofacitinib use and an increased risk of NMSC in other therapeutic indications [37].

In 2022, Sandborn et al. published the latest results of the OCTAVE Open trial, an open-label, long-term extension study with up to 7.0 years of treatment with tofacitinib in moderately to severely active UC [38]. Malignancies were rare, and the safety profile remained consistent with prior analyses conducted within the OCTAVE clinical program [38].

Another significant result is that extended induction therapy with tofacitinib at a dose of 10 mg twice daily (b.i.d.) for a total of 16 weeks did not increase the risk of adverse events, including the development of malignancies [39].

The study by Panés et al. reports the latest data from the global tofacitinib UC clinical program with up to 9.2 years of follow-up [40]. This analysis included patients receiving tofacitinib 5 or 10 mg twice daily (b.i.d.) from completed phase II/III placebo-controlled studies; the open-label, long-term extension study; and a randomized phase IIIb/IV study. Malignancies (excluding NMSC) were reported in 29 patients, with an incidence rate of 0.88 (95% CI, 0.59–1.26) [40]. Notably, 5 cases were observed in the group receiving tofacitinib 5 mg twice daily and 24 cases in the group receiving tofacitinib 10 mg twice daily, corresponding to incidence rates of 0.54 (95% CI, 0.18–1.26) and 1.01 (95% CI, 0.65–1.51), respectively [40]. The reported malignancies (excluding NMSC) included one case each of acute myeloid leukemia, Bowen’s disease, Epstein–Barr virus-associated lymphoma, essential thrombocythemia, hepatic angiosarcoma, leiomyosarcoma, esophageal adenocarcinoma, penile dysplasia, renal cell carcinoma, vulvar cancer, and prostate cancer; two cases each of cervical dysplasia, cholangiocarcinoma, diffuse large B-cell lymphoma, lung cancer, and malignant melanoma; three cases of breast cancer; and five cases of colorectal cancer [40]. NMSC cases were reported in 23 patients, corresponding to an incidence rate of 0.71 (95% CI, 0.45–1.07) [40]. Of these, 6 cases were reported in the group receiving tofacitinib 5 mg twice daily and 17 in the group receiving tofacitinib 10 mg twice daily, with respective incidence rates of 0.66 (95% CI, 0.24–1.43) and 0.73 (95% CI, 0.43–1.17) [40]. Among these cases, 17 patients were diagnosed with basal cell carcinoma and 11 with squamous cell carcinoma [40].

The study emphasizes that the risk of malignancies was not found to be elevated, even among patients over 50 years of age or those with a history of smoking, despite these subgroups representing only a small proportion of the UC clinical program [40]. However, the analyzed studies were not designed to be of sufficient size or duration to adequately assess rare events or those with long latency periods [40].

A recent systematic review and meta-analysis conducted by Bezzio et al. evaluated the overall cancer risk associated with tofacitinib therapy in various diseases and concluded that there is no evidence of an increased neoplastic risk linked to its use [41].

Real-world data provide reassuring evidence regarding the safety profile of tofacitinib in patients with UC. The REMIT-UC study, a Canadian multicenter real-world cohort study, included 334 consecutive adult outpatients with UC treated with tofacitinib, followed up for a total duration of 375 patient-years. A total of three malignancies were reported, corresponding to an incidence rate of 0.8 events per 100 patient-years (95% CI: 0.2–2.3): one case of Kaposi sarcoma in a 50-year-old man with human immunodeficiency virus (HIV), one case of an incidentally detected small bowel neuroendocrine tumor in a 28-year-old woman, and one case of multiple myeloma in a 35-year-old man [42].

In addition, Chaparro et al. conducted a multicenter real-world evidence study on tofacitinib in patients with ulcerative colitis, involving 408 individuals across Spain with a follow-up period of three years. One case of neoplasia was reported (0.2%) but was not attributed to tofacitinib. Only two cases of NMSC were reported, corresponding to an incidence rate of 0.47 (95% CI, 0.11–1.87), with neither case deemed related to tofacitinib therapy [43] [Table 1].

## 4. Filgotinib

Filgotinib, a JAK1-selective inhibitor, has been evaluated in IBD patients and approved for UC through the SELECTION trial.

The SELECTION trial highlighted the efficacy of filgotinib in moderate-to-severe UC, with a favorable safety profile and no significant increase in malignancy risks during the study period, with a safety profile comparable to the placebo [44]. NMSC cases were observed in three patients during the induction studies (one in the placebo group, 0.4%, and two in the filgotinib group, 0.4%) and in one patient during the maintenance study (0.6%) [44]. All patients who developed NMSC had a history of treatment with thiopurines [44]. Malignancies were reported in three patients: one case of colon cancer in induction study A (filgotinib 100 mg, 0.2%), one case of breast cancer in induction study B (filgotinib 200 mg, 0.2%), and one case of malignant melanoma in the maintenance study (filgotinib 200 mg, 0.5%) [44]. However, the limited duration of this trial leaves uncertainties regarding long-term outcomes.

The interim analysis of the SELECTIONLTE study, which included data from up to approximately 4 years of filgotinib treatment, indicated no increased risk of malignancies with prolonged use [45]. Adverse event rates were comparable between the filgotinib 100 mg (FIL100) group and the filgotinib 200 mg (FIL200) group [45]. In the FIL200 group, the exposure-adjusted incidence rates (EAIRs) for both malignancies excluding NMSC were below 1.0 per 100 patient-years of exposure (cPYE) [45]. In the FIL100 group, the EAIR for NMSC was also below 1.0 per 100 cPYE, while the EAIR for malignancies excluding NMSC was 2.0 per 100 cPYE [45]. However, it is important to note that these analyses did not specifically focus on the subgroup of at-risk patients as defined by the Pharmacovigilance Risk Assessment Committee (e.g., current malignancy or history of malignancy) [46].

Another study assessing the safety profile of filgotinib was the phase II MANTA trial, which aimed to evaluate the potential effects of filgotinib on semen parameters and changes in sex hormones in men with inflammatory diseases [47]. The study compared filgotinib 200 mg with a placebo over a treatment period of up to 65 weeks (13 weeks of induction followed by 52 weeks of extension phase) [47]. No cases of malignancies or NMSC were reported during the study [47].

The first data on the real-world efficacy and safety of filgotinib in UC were reported by Gros et al. in a real-world cohort study including 91 patients [48]. Following a median follow-up period of 39 weeks, no cases of malignancy were reported among the patients. The median age of the cohort was 42 years [48].

The most recent real-world assessment study conducted by Young et al. across nine centers in the UK included a total of 286 patients, with a median follow-up duration of 229 days (IQR 113–324). The only reported case of malignancy involved a 52-year-old male patient who was also diagnosed with primary sclerosing cholangitis and was found to have liver adenocarcinoma of unknown primary origin four months after initiating treatment with filgotinib [49] [Table 2].

Overall, filgotinib appears to be a safe molecule, though longer-term studies are needed to confirm this.

## 5. Upadacitinib

Upadacitinib, a JAK1-selective inhibitor, has shown promising results in IBD treatment. The U-ACCOMPLISH [50], U-ACHIEVE Induction [51] and Maintenance [52], U-EXCEL, U-EXCEED, and U-ENDURE [53] studies demonstrated that upadacitinib is effective for both induction and maintenance of remission in UC and Crohn’s disease. Notably, these trials reported no significant increase in malignancy rates during the study period in comparison to the placebo.

U-ACCOMPLISH is one of two phase III induction trials designed to evaluate the safety and efficacy of upadacitinib 45 mg once daily in adult patients with UC. This multicenter, randomized, double-blind, placebo-controlled study enrolled patients with moderately to severely active disease who had experienced an inadequate response; loss of response; or intolerance to aminosalicylates, immunosuppressants, corticosteroids, and/or biologic therapies. Patients were randomized in a 2:1 ratio to receive upadacitinib 45 mg once daily or a placebo for a duration of 8 weeks. No cases of malignancies or NMSC were reported during the study period [50].

The other phase III induction trial, U-ACHIEVE Induction, is a multicenter, double-blind, with a placebo-controlled design, in which patients with moderately to severely active UC were randomized in a 2:1 ratio to receive upadacitinib 45 mg once daily or a placebo for 8 weeks. Among the total of 474 patients enrolled, no cases of malignancies or NMSC were reported [51].

In the U-ACHIEVE Maintenance trial, 681 patients with previous moderately to severely active UC who had achieved a clinical response with upadacitinib 45 mg/day during the induction trials (the phase IIb U-ACHIEVE Induction and U-ACCOMPLISH trials) were enrolled and randomized to receive a placebo, upadacitinib 30 mg/day, or upadacitinib 15 mg/day [52]. Among the participants, one malignancy (excluding NMSC) was reported in the placebo group, one in the upadacitinib 15 mg/day group (invasive breast carcinoma), and two in the upadacitinib 30 mg/day group (colon adenocarcinoma and small cell carcinoma of the prostate), corresponding to incidence rates of 0.7, 0.5, and 0.9 events per 100 patient-years, respectively [52]. To assess the safety of upadacitinib in patients at higher risk of malignancies, data from the safety population were analyzed for baseline risk factors that could increase the likelihood of these events [52]. All three patients with malignancies had at least one relevant known risk factor, and the time to onset of these events from the first dose of upadacitinib was approximately one year [52]. None of the malignancies (excluding NMSC) reported with upadacitinib were deemed by the investigators to have a reasonable likelihood of being related to the treatment [52]. This conclusion was supported by the short interval between the initiation of upadacitinib and the onset of malignancies, suggesting temporal implausibility for upadacitinib to play a causative role in the oncogenesis of the reported cases [52]. NMSC was observed only in three patients receiving upadacitinib 30 mg once daily (two out of the three cases were in patients ≥70 years old), with no cases in patients receiving upadacitinib 15 mg once daily or the placebo [52].

The long-term safety profile of upadacitinib will be further assessed using data from the ongoing U-ACTIVATE long-term extension study (NCT03006068) [54].

For CD, two phase III studies, U-EXCEL and U-EXCEED, were conducted to evaluate the efficacy of upadacitinib 45 mg versus the placebo over 12 weeks as induction therapy in patients with moderate-to-severe CD [53]. In a total of 526 patients for U-EXCEL and 495 patients for U-EXCEED, no cases of malignancies were reported [53].

A total of 502 patients who achieved a clinical response during these induction trials were subsequently enrolled in the U-ENDURE trial, which assessed maintenance therapy with a placebo, upadacitinib 30 mg daily, or upadacitinib 15 mg daily over a 52-week period [53]. No cases of malignancies were reported in the two induction trials [53]. However, in the U-ENDURE trial, one case of metastatic ovarian cancer was reported in the upadacitinib 15 mg group, and two cases of malignancies (colon cancer and invasive lobular breast cancer) were reported in the upadacitinib 30 mg group [53]. These malignancies were diagnosed on trial days 159, 8, and 183 after the first dose of maintenance treatment, respectively [53]. The authors concluded that although patients with CD receiving immunosuppressive therapy may have a modestly increased risk of developing cancer, the small number of cancer cases observed in the U-ENDURE trial, coupled with the limited treatment exposure, the timing of occurrence, and the absence of a discernible pattern in the types of cancers reported, did not support a definitive conclusion of an increased cancer risk associated with upadacitinib [53].

Garcia et al. conducted a Spanish Nationwide Study (U-REAL Study) to evaluate the effectiveness and safety of upadacitinib in CD and UC in real life. The cohort included 100 patients, comprising 68 with Crohn’s disease and 32 with ulcerative colitis, who had previously received a median of four advanced therapies. With a median follow-up duration of 7.6 months (interquartile range 4.6–14), no cases of malignancies were reported [55].

The real-world effectiveness and safety of upadacitinib in patients with CD was further evaluated by Devi et al. through a retrospective analysis conducted across nine centers in the United States. The study included adult patients with active luminal CD who received upadacitinib 45 mg once daily as induction therapy, followed by 30 mg daily maintenance therapy. The co-primary endpoints were clinical remission at week 12 and endoscopic remission at 6 months. A total of 334 CD patients were included in the analysis. Notably, no cases of malignancies were reported during the study period [56] [Table 3].

However, the limited duration of these studies precluded definitive conclusions about long-term cancer risks. Long-term registries and post-marketing surveillance will be critical in assessing upadacitinib’s safety profile.

## 6. Discussion

JAK inhibitors present a novel therapeutic option in the management of IBDs, with a reassuring safety profile.

The issue of an increased risk of cancer associated with immunomodulatory and immunosuppressive therapies for IBDs has already been raised. Thiopurines and corticosteroids, long-standing treatments for IBDs, have well-documented associations with malignancies, such as non-Hodgkin lymphoma and skin cancers [57,58]. Although anti-TNF agents were previously suspected to be associated with an increased oncogenic risk [59,60], in particular lymphoma and NMSC, such a hypothesis has not been confirmed by subsequent evidence. According to ECCO guidelines, there is no evidence of an overall increased risk of cancer in patients with IBDs treated with anti-TNF monotherapy [61]. In line with ECCO recommendations, skin cancer surveillance and sun protection strategies should be encouraged based on individual risk factors, although additional screening beyond standard population guidelines is not currently warranted [61].

It is crucial to underline that chronic inflammatory conditions such as IBDs are well documented to increase the risk of malignancies [62]. This association is primarily attributed to persistent inflammation, which fosters a pro-tumorigenic environment through mechanisms such as oxidative stress, the chronic activation of cytokine pathways, and DNA damage [62]. Additionally, immune dysregulation in these diseases may impair the body’s natural tumor surveillance mechanisms [62]. Notably, therapeutic interventions aimed at suppressing chronic inflammation have been shown to reduce cancer risk in such patients [63]. For example, studies have reported that anti-TNF agents not only alleviate inflammation but also decrease the incidence of certain inflammation-associated malignancies, such as colorectal cancer in patients with IBDs [63]. However, the advent of JAK inhibitors has raised important questions about their long-term safety, particularly regarding cancer risk, given the central role of the JAK-STAT pathway in immune regulation and cellular growth [32]. Despite these theoretical concerns, evidence from clinical trials and real-world data suggest that JAK inhibitors can be used safely in IBD populations, particularly when patient-specific risk factors are considered [64].

Safety concerns regarding tofacitinib oncogenic potential emerged following the publication of the ORAL Surveillance study [31]. However, it is important to recognize that the ORAL Surveillance study was conducted in a unique cohort with baseline risk factors not representative of the typical IBD population, which tends to be younger and less likely to exhibit smoking-related malignancies [31]. The evidence to date does not conclusively establish a direct oncogenic risk for tofacitinib in IBD patients, particularly in those without significant baseline risk factors such as age, smoking history, or prior malignancy. While the ORAL Surveillance findings have driven cautionary approaches, the extrapolation of data from RA to IBDs should be approached carefully. Until further evidence emerges, tofacitinib appears to be a safe option for younger IBD patients, particularly in the absence of major risk factors for cancer. Moreover, real-world evidence and meta-analyses have suggested that the malignancy risk associated with tofacitinib in IBD patients is not significantly elevated compared to other therapeutic options or placebo treatment [41].

Clinical trials and real-world data for filgotinib and upadacitinib in IBD have not identified an increased risk of malignancy thus far [48,49,55,56]. These findings suggest a favorable safety profile for both agents, although their relatively recent introduction and the limited duration of follow-up necessitate cautious interpretation. Long-term observational studies and post-marketing surveillance are necessary to fully characterize their safety profiles.

Overall, there is no evidence that the overall risk of cancer is increased in patients with IBD treated with JAK inhibitors [61]. The lack of prospective studies specifically designed to evaluate the risk of malignancies with JAK inhibitors in IBD patients represents a significant knowledge gap. While retrospective analyses and meta-analyses provide useful insights, prospective, long-term studies are essential to accurately assess cancer risk. Such studies should include diverse patient populations, stratified by age, baseline risk factors, and disease severity, to provide robust and generalizable data. Incorporating biomarkers for cancer risk could also enhance the predictive value of these studies, guiding clinical decision-making and optimizing patient outcomes. At the same time, real-world evidence and post-marketing surveillance play an essential role in understanding the long-term safety of JAK inhibitors. While randomized clinical trials provide valuable insights, they often exclude patients with significant comorbidities or those at higher risk of malignancies. Registries and observational studies capturing data from diverse patient populations are vital to identifying rare adverse events, including cancer. Post-marketing surveillance data can bridge the gap between clinical trial findings and real-world outcomes, providing a more comprehensive understanding of the risk profile of JAK inhibitors in routine clinical practice. Until such data are available, clinicians should remain vigilant, particularly in patients with established risk factors for malignancy.

For patients with a high baseline risk of cancer—such as smokers, individuals with a personal or family history of malignancy, or those with previous exposure to immunosuppressive therapies—alternative treatment strategies may be more appropriate. Conversely, younger patients with fewer risk factors may benefit from the potent anti-inflammatory effects of JAK inhibitors without significant concern for malignancy.

In clinical practice, regular cancer screening remains essential as part of a comprehensive safety strategy. This includes adherence to endoscopic surveillance protocols, particularly in patients with long-standing ulcerative colitis or colonic Crohn’s disease, given their established risk for colorectal cancer [65]. In addition, dermatological monitoring, including periodic full-body skin examinations, is recommended to detect early signs of non-melanoma and melanoma skin cancers. Female patients should also undergo routine gynecological surveillance, including cervical cytology (Pap test), in accordance with national screening guidelines. Furthermore, HPV vaccination may be considered, especially in younger patients or those with additional risk factors, as part of a broader preventive care approach [66].

Ultimately, a patient-centered management strategy, based on shared decision-making and individualized risk assessment, is essential to ensure the safe and effective use of JAK inhibitors in the treatment of inflammatory bowel disease.

## 7. Conclusions

JAK inhibitors have emerged as effective therapeutic options for the management of IBD. While IBD itself increases the baseline risk of cancer, the effective suppression of chronic inflammation through therapies such as JAK inhibitors has the potential to mitigate this risk. Concerns regarding the oncogenic potential of JAK inhibitors, particularly tofacitinib, have been raised based on findings from the ORAL Surveillance study. However, these findings may not apply to the IBD population. Emerging data for selective JAK inhibitors, such as upadacitinib and filgotinib, suggest a favorable safety profile, though further research is warranted. As with other immunomodulatory therapies, JAK inhibitors should be used with appropriate clinical judgment, taking into account individual patient risk factors and applying standard monitoring strategies. Current evidence does not indicate the need for heightened oncologic vigilance beyond what is routinely applied with other therapeutic classes used in IBD treatment.

## Figures and Tables

**Table 1:** Tofacitinib and risk of cancer in IBD patients.

Tofacitinib						
Study	Year	Population	Duration of the Study	Groups	Cancer (Nr/Total)	NMSC (Nr/Total)
OCTAVE 1 [35]	2016	UC	8 weeks	10 mg	-	1/476 (0.2%)
OCTAVE 2 [35]	2016	UC	8 weeks	10 mg	-	1/429 (0.2%)
OCTAVE Sustain [36]	2017	UC	52 weeks	Placebo	1/198 (0.5%)	1/198 (0.5%)
				5 mg	-	-
				10 mg	-	3/196 (1.5%)
OCTAVE Open [38]	2022	UC	Up to 7 years	5 mg	7/175 (4%)	8/175 (4.6%)
				10 mg	18/796 (2.3%)	14/796 (1.8%)
Global Clinical Program (Panés et al.) [40]	2024	UC	Up to 9.2 years	5 mg	5/219 (2.3%)	6/219 (2.7%)
				10 mg	24/938 (2.7%)	17/938 (1.9%)
REMIT-UC [42]	2022	UC	365 patient-years	NA	3/334 (0.8%)	-
Chaparro et al. [43]	2023	UC	3 years	NA	1/408 (0.2%)	2/408 (0.47%)

UC: ulcerative colitis; NMSC: non-melanoma skin cancer; NA: not applicable.

**Table 2:** Filgotinib and risk of cancer in IBD patients.

Filgotinib						
Study	Year	Population	Duration of the Study	Groups	Cancer (Nr/Total)	NMSC (Nr/Total)
SELECTION [44]	2021	UC	11 weeks (Induction A and B)	Placebo	-	1/279 (0.4%)
				100 mg	1/562 (0.2%)	-
				200 mg	1/507 (0.2%)	2/507 (0.4%)
			52 weeks (Maintenance)	Placebo	-	-
				100 mg	-	1/179 (0.6%)
				200 mg	1/202 (0.5%)	-
SELECTIONLTE [45]	2024	UC	Up to 4 years	100 mg	6/160 (3.7%)	1/160 (0.6%)
				200 mg	10/873 (1.1%)	12/873 (1.4%)
MANTA [47]	2022	Male UC, CD	13 + 52 weeks	Placebo	-	-
				200 mg	-	-
Gros et al. [48]	2025	UC	39 weeks	200 mg	-	-
Young et al. [49]	2025	UC		200 mg	1/286 (0.3%)	-

UC: ulcerative colitis; CD: Crohn’s disease; NMSC: non-melanoma skin cancer.

**Table 3:** Upadacitinib and risk of cancer in IBD patients.

Upadacitinib						
Study	Year	Population	Duration of the Study	Groups	Cancer (Nr/Total)	NMSC (Nr/Total)
U-ACCOMPLISH [50]	2021	UC	8 weeks	Placebo	-	-
				45 mg	-	-
U-ACHIEVE Induction [51]	2021	UC	8 weeks	Placebo	-	-
				45 mg	-	-
U-ACHIEVE Maintenance [52]	2023	UC	52 weeks	Placebo	1/245 (0.7%)	-
				15 mg	1/250 (0.5%)	-
				30 mg	2/251 (0.9%)	3/251 (1.4%)
U-EXCEED [53]	2021	CD	12 weeks	Placebo	-	-
				45 mg	-	-
U-EXCEL [53]	2022	CD	12 weeks	Placebo	-	-
				45 mg	-	-
U-ENDURE [53]	2023	CD	52 weeks	Placebo	-	-
				15 mg	1/221 (0.7%)	-
				30 mg	2/229 (1.2%)	-
Garcia et al. [55]	2024	CD, UC	Median follow-up of 7.6 months	15 mg	-	-
				30 mg	-	-
				45 mg	-	-
Devi et al. [56]	2025	CD	6-month follow-up	Induction 45 mg, maintenance 30 mg	-	-

UC: ulcerative colitis; CD: Crohn’s disease; NMSC: non-melanoma skin cancer.

## Data Availability

No new data were created or analyzed in this study. Data sharing is not applicable to this article.
